# Perceived exposure to hazardous working conditions in Germany

**DOI:** 10.17886/RKI-GBE-2017-132

**Published:** 2017-12-13

**Authors:** Lars Eric Kroll, Stephan Müters, Maria Schumann, Lampert Thomas

**Affiliations:** Robert Koch Institute, Department of Epidemiology and Health Monitoring, Berlin

**Keywords:** OCCUPATIONAL HEALTH, WORKING CONDITIONS, SOCIAL INEQUALITY, HEALTH MONITORING, GERMANY

## Abstract

Data on the prevalence of perceived exposure to hazardous working conditions were gathered for the GEDA 2014/2015-EHIS study using a simple graduated question. Approximately one-fifth of people in employment state that they face serious or very serious occupational health hazards. However, women (18.6%) are significantly less likely to have this perception than men (27.0%). The differences between women and men can be explained by the variation in working hours and by the continued gender specific division of the labour market (segregation). There are pronounced differences among men with regard to educational and vocational qualifications, with lower qualified men viewing their employment as posing a higher risk to their health than higher qualified men; no similar differences exist between women. Finally, perceived health risks are highest among women and men in the passenger and freight transport sectors. The results of this study underline the importance of occupational safety and workplace health promotion.

## Introduction

Occupational health hazards result from the presence of physical or psychological stress at work, and the risk of accident and injury in the workplace or while traveling to work [1, 2]. Technological progress and economic globalisation are factors promoting a more intensified form of labour: processes are accelerating in manufacturing, the service industry and communications; permanent forms of employment are becoming scarcer, and the demands placed on people in the workplace in terms of mobility and accessibility are increasing [3, 4].

Ergonomics uses the concept of stress and strain to describe the health burdens that arise from work [4, 5]. Stress is viewed as placing a strain on the health of the workforce. This is especially important when the stress levels or the duration during which people are exposed to stress exceeds their physical or mental capacities and particularly when this stress cannot be offset by technical or organisational measures. In general, low-skilled workers are most frequently exposed to stress, and are more likely to be exposed to higher levels of stress [6].

Many studies emphasise the complex interactions that exist between working conditions, employment and health [7]. Physical and psychological stress in the workplace are closely linked to disease and illness [4, 8-10], accidents at work [11], incapacity to work, [12, 13] early retirement [14, 15] and higher mortality rates [16, 17]. Moreover, the association between work-related stress and health exists in every European country and is largely independent of any specific form of state welfare provision [18]. This leads to the view within occupational safety and occupational health management that working conditions need to be improved because they have consequences for health, lead to absences from work, and produce costs [19].

**GEDA 2014/2015-EHIS**

**Data holder:** Robert Koch Institute

**Aims:** To provide reliable information about the population’s health status, health-related behaviour and health care in Germany, with the possibility of a European comparison

**Method:** Questionnaires completed on paper or online

**Population:** People aged 18 years and above with permanent residency in Germany

**Sampling:** Registry office sample; randomly selected individuals from 301 communities in Germany were invited to participate

**Participants:** 24,016 people (13,144 women; 10,872 men)

**Response rate:** 26.9%

**Study period:** November 2014 - July 2015

**Data protection:** This study was undertaken in strict accordance with the data protection regulations set out in the German Federal Data Protection Act and was approved by the German Federal Commissioner for Data Protection and Freedom of Information. Participation in the study was voluntary. The participants were fully informed about the study’s aims and content, and about data protection. All participants provided written informed consent.

More information in German is available at www.geda-studie.de

## Indicator

As part of the GEDA 2014/2015-EHIS study, data was gathered for the indicator ‘perceived hazardous working conditions’ using a questionnaire that the respondents completed on paper or online. There are many different indicators that can be used to measure occupational health hazards but they are often too extensive to be taken into account by health surveys. In the GEDA 2014/2015-EHIS study, respondents in employment, therefore, were asked to assess the impact of their work on their health. This was done using the following question: ‘Do you think that your work poses a risk to your health?’ The possible answers were: ‘[Work poses a] very serious risk [to my health]’, ‘a serious risk’, ‘a moderate risk’ or ‘no risk at all’. The data gathered from the first two answers were combined, which means that the following refers to respondents who viewed their work as posing a ‘very serious’ or ‘serious’ risk to their health.

The analyses conducted for the GEDA study are based on data gathered from 14,265 participants in employment aged between 18 and 64 (7,761 women; 6,504 men) who provided valid data on hazardous working conditions. The calculations were carried out using a weighting factor that corrected the sample for deviations from Germany’s population structure (as of 31 December 2014) in terms of gender, age, district type and level of education. The district type reflects the degree of urbanisation and accounts for the regional distribution in Germany. The International Standard Classification of Education (ISCED) was used to classify the responses provided on educational level [20]. A detailed description of the methodology used for GEDA 2014/2015-EHIS can be found in Lange et al. 2017 [21], as well as in the article German Health Update: New Data for Germany and Europe, which was published in issue 1/2017 of the Journal of Health Monitoring.

## Results and discussion

The results of the GEDA 2014/2015-EHIS study show that 23.0% of people in employment state that they face serious or very serious occupational health hazards. These rates are higher than those identified by earlier GEDA studies, where just under 16.8% of the workforce in 2010 and 20.9% in 2012 reported that they faced serious or very serious occupational health risks. However, in all of these studies, women had lower rates of perceived occupational health risks than men. In 2014, 18.6% of women and 27.0% of men reported that they viewed their employment as posing a risk to their health (Table 1). Even after controlling for age differences and differences in the extent of employment, a significant increase in perceived hazardous working conditions occurred between 2010 and 2014 among women and men.

The proportion of women who viewed their working conditions as posing a serious or very serious risk to their health remains relatively stable among women with increasing age. In contrast, significantly lower rates of men age between 18 and 29 reported that they faced occupational health risks compared with men in the two older aged groups (Table 1). There are also pronounced differences among men with regard to educational and vocational qualifications, with low qualified men reporting higher levels of risk. There are no similar significant differences in this regard among women. While interpreting the results, it is important to take into account the fact that the sector, type and extent of employment (such as part-time work) are of particular importance with regard to the extent to which people are exposed to occupational health hazards. For example, low qualified men often work under physically stressful conditions; this is not the case for women to the same extent [6].

In order to statistically control for age structure and extent of employment when comparing the perceptions of occupational health hazards in various industries, a model was used to develop an estimate for a hypothetical 40-year-old full-time employee (39 hours a week). The predicted prevalences are shown in Table 2. No results are displayed for sectors with a sample size of less than 50 women or men. The results demonstrate that particularly high numbers of women in the passenger and freight transport sector, as well as in health and social work, believe that their work poses a risk to their health. The highest proportion of men with perceived occupational health risks are also employed in passenger and freight transport, but also in the construction industry. A significantly smaller proportion of people working in service provision – such as in the financial sector and freelance professionals – view their employment as posing a risk to their health. The differences identified according to industry correspond with data from statutory health insurers, which also demonstrate higher rates of illness-related absence among people employed in the transport sector [19].

In Germany, employers have a statutory obligation to ensure the safety and to protect the health of their employees in the workplace. In addition, the Preventative Health Care Act, which was passed in 2015, made health promotion in the workplace a focus for health insurers. Nevertheless, almost a quarter of people in employment continue to view their work as hazardous to their health, and this tendency is increasing. These results emphasise the continuing importance of health promotion and occupational safety in protecting the health of the population.

## Key statements

- Approximately one fifth of people in employment believe that they face serious or very serious health hazards in their workplace; women are significantly less likely to have this perception than men.
- Perceived occupational health hazards rose from 16.8% in 2010 to 23.0% in 2014.
- High levels of perceived occupational health hazards are reported by women and men working in the passenger and freight transport sectors irrespective of age or the extent of their employment.
- The large numbers of people facing high levels of subjective burdens at work underlines the continued importance of occupational health and workplace health promotion.

## Figures and Tables

**Table 1:** The prevalence of perceived occupational health hazards (‘serious’ and ‘very serious’) according to gender, age and educational level (n=7,761 women; n=6,504 men) Source: GEDA 2014/2015-EHIS

Women	%	(95% CI)	Men	%	(95% CI)
**Women (total)**	**18.6**	**(17.5-19.7)**	**Men (total)**	**27.0**	**(25.6-28.5)**
**18-29 Years**	19.9	(17.5-22.6)	**18-29 Years**	22.9	(19.8-26.2)
Low education	18.0	(11.5-27.0)	Low education	20.4	(14.1-28.6)
Medium education	21.1	(18.0-24.6)	Medium education	26.6	(22.5-31.1)
High education	17.4	(13.4-22.2)	High education	10.8	(7.3-15.5)
**30-44 Years**	17.3	(15.6-19.3)	**30-44 Years**	28.3	(25.9-30.9)
Low education	12.2	(7.6-19.1)	Low education	41.1	(32.1-50.7)
Medium education	18.1	(15.7-20.8)	Medium education	31.4	(28.0-35.0)
High education	17.6	(14.9-20.8)	High education	18.4	(15.8-21.4)
**45-64 Years**	18.8	(17.3-20.4)	**45-64 Years**	27.9	(26.1-29.8)
Low education	18.0	(14.2-22.5)	Low education	34.5	(29.5-39.7)
Medium education	18.5	(16.7-20.5)	Medium education	31.9	(29.2-34.8)
High education	20.4	(17.9-23.1)	High education	19.2	(17.2-21.4)
**Total (women and men)**	**23.0**	**(22.1-24.0)**	**Total (women and men)**	**23.0**	**(22.1-24.0)**

CI=confidence interval

**Table 2:** Perceived occupational health hazards according to gender and sector; predicted prevalence for a 40-year-old full-time employee (n=7,276 women; n=6,138 men) Source: GEDA 2014/2015-EHIS

Sector	Women		Men	
	%	(95% CI)	%	(95% CI)
Agriculture, forestry and fisheries	34.9	(20.1-49.6)	31.8	(20.5-43.1)
Manufacturing industries/Goods manufacturing	19.3	(15.1-23.6)	30.8	(27.5-34.1)
Energy supply	5.6	(0.0-12.4)	28.6	(19.0-38.1)
Construction	10.7	(3.7-17.8)	45.1	(39.7-50.5)
Wholesale and retail; Car mechanics	21.1	(16.5-25.6)	24.1	(19.3-28.9)
Passenger and freight transport; Warehousing, courier services	36.9	(25.6-48.2)	46.0	(39.0-53.1)
Catering, gastronomy	33.9	(25.4-42.3)	31.2	(18.7-43.6)
Information and communication	13.4	(8.4-18.3)	17.0	(12.6-21.5)
Banking/financial and insurance service providers	16.1	(11.6-20.6)	21.8	(15.3-28.3)
Real estate and housing	18.0	(6.2-29.9)	17.1	(5.2-29.0)
Freelance professionals	16.3	(10.8-21.8)	22.2	(16.0-28.4)
Other financial services	16.7	(9.5-23.9)	30.3	(19.9-40.8)
Administration	19.3	(15.0-23.7)	25.1	(20.7-29.6)
Schooling and teaching	28.8	(24.8-32.8)	18.3	(12.8-23.7)
Health and social services	36.6	(33.1-40.2)	35.1	(28.8-41.5)
Other people-related services	24.4	(15.3-33.6)	32.9	(22.0-43.8)
Art, entertainment, sports and recreation	15.2	(5.0-25.4)	17.8	(7.3-28.2)

CI=confidence interval

Results for sectors with less than n=50 women or men in the sample are not shown
